# Shared decision making in pregnancy in inflammatory bowel disease: design of a patient orientated decision aid

**DOI:** 10.1186/s12876-021-01853-y

**Published:** 2021-07-30

**Authors:** Astrid-Jane Williams, Neda Karimi, Radha Chari, Susan Connor, Mary A. De Vera, Levinus A. Dieleman, Tawnya Hansen, Kathleen Ismond, Rshmi Khurana, Dawn Kingston, Katie O’Connor, Daniel C. Sadowski, Flora Fang-Hwa, Eytan Wine, Yvette Leung, Vivian Huang

**Affiliations:** 1 Department of Gastroenterology and Hepatology, Liverpool Hospital, Elizabeth St, Liverpool, Sydney, NSW 1871 Australia; 2 South Western Sydney Clinical School, The University of New South Wales, Sydney, Australia; 3 Ingham Institute for Applied Medical Research, Sydney, NSW Australia; 4 University of British Columbia, Vancouver, BC Canada; 5 University of Alberta, Edmonton, AB Canada; 6 University of Calgary, Calgary, AB Canada; 7 Mount Sinai Hospital, Sinai Health System, University of Toronto, Toronto, ON Canada

**Keywords:** Inflammatory bowel disease, Pregnancy, Conception, Decision making

## Abstract

**Background:**

Research has indicated a lack of disease-specific reproductive knowledge among patients with Inflammatory Bowel Disease (IBD) and this has been associated with increased “voluntary childlessness”. Furthermore, a lack of knowledge may contribute to inappropriate medication changes during or after pregnancy. Decision aids have been shown to support decision making in pregnancy as well as in multiple other chronic diseases. A published decision aid for pregnancy in IBD has not been identified, despite the benefit of pre-conception counselling and patient desire for a decision support tool. This study aimed to develop and test the feasibility of a decision aid encompassing reproductive decisions in the setting of IBD.

**Methods:**

The International Patient Decision Aid Standards were implemented in the development of the Pregnancy in IBD Decision Aid (PIDA). A multi-disciplinary steering committee was formed. Patient and clinician focus groups were conducted to explore themes of importance in the reproductive decision-making processes in IBD. A PIDA prototype was designed; patient interviews were conducted to obtain further insight into patient perspectives and to test the prototype for feasibility.

**Results:**

Issues considered of importance to patients and clinicians encountering decisions regarding pregnancy in the setting of IBD included fertility, conception timing, inheritance, medications, infant health, impact of surgery, contraception, nutrition and breastfeeding. Emphasis was placed on the provision of preconception counselling early in the disease course. Decisions relating to conception and medications were chosen as the current focus of PIDA, however content inclusion was broad to support use across preconception, pregnancy and post-partum phases. Favourable and constructive user feedback was received.

**Conclusions:**

The novel development of a decision aid for use in pregnancy and IBD was supported by initial user testing.

## Background

Inflammatory Bowel Disease (IBD) includes chronic conditions of the intestines namely Crohn’s disease (CD) and ulcerative colitis (UC). IBD is increasingly diagnosed at younger ages and is usually managed with medications and/or surgery [1]. It is known that the lack of IBD-specific reproductive knowledge among patients has been associated with increased “voluntary childlessness”, with reported rates of 18% and 14% in patients with CD and UC respectively compared with 6.2% in the general population [2, 3]. Furthermore, a lack of patient and clinician knowledge may contribute to inappropriate medication cessation during attempts at conception or pregnancy and increase the risk of flares, despite the expanding data supporting drug safety in pregnancy [4–7]. In particular, there is increasing evidence supporting the safety of biologics. With appropriate information provided to both patients and clinicians, it is anticipated that a greater proportion of patients will receive necessary IBD therapy that has not otherwise been prescribed or adhered to due to misinformation, with resultant optimization of maternal and foetal outcomes [8].

Active IBD during preconception adversely impacts fertility and increases the risk of active disease throughout pregnancy. Thus, it is recommended that patients be in remission before attempting to conceive [9, 10]. Several studies have demonstrated that IBD activity during pregnancy can adversely impact outcomes. For example, a prospective Danish cohort study of women with a history of moderate to severely active IBD reported that disease activity was associated with an increased risk of low birth weight (adjusted odds ratio 2.05; 95% confidence interval: 0.37–11.35) and preterm birth (2.64; 1.14–11.36) [11]. It is also known that active IBD is associated with an increased risk of miscarriage [12, 13].

A significant proportion of women with IBD are of child-bearing age and therefore, a decision aid focusing on reproductive decisions in the context of having IBD has the potential to have significant impact for both patients and clinicians. A Canadian survey study conducted between 2012 and 2014 of women with IBD and clinicians involved in the treatment of patients with IBD confirmed a lack of reproductive knowledge specific to IBD and a desire for more information [14]. While there are existing evidence-based decision aids designed to support decision making in pregnancy in general, as well as in multiple other chronic diseases [15–17], a review of the existing literature has not identified such a resource for pregnancy in IBD. This is despite studies indicating the benefit of pre-conception counselling and patient desire for education and a decision support tool [14, 18–20].

Accordingly, we ascertained issues considered of importance to patients and clinicians encountering decisions regarding pregnancy in the setting of IBD to guide the design of a patient-focused decision aid intended for use in preconception, pregnancy and post-partum phases. Following identification of pertinent issues, an electronic decision aid was created, with the subsequent study aim to evaluate the feasibility of the decision aid using a user-centered approach.

## Methods

### Overview

**Fig. 1:**
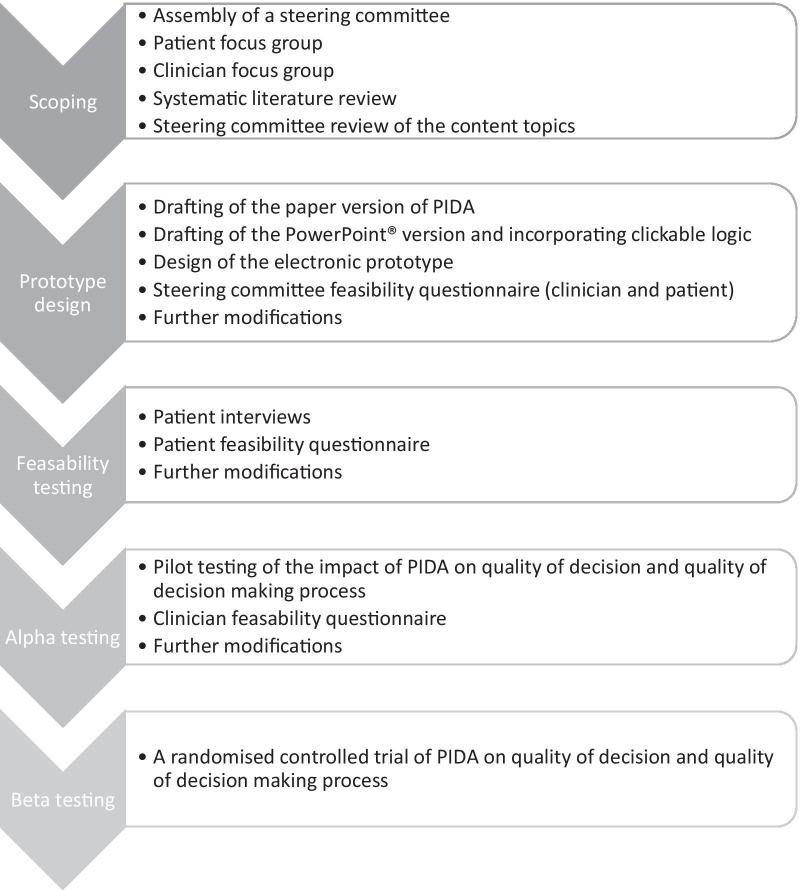
Flow diagram of decision aid development and evaluation. PIDA: Pregnancy in IBD decision aid

The International Patient Decision Aid Standards (IPDAS) guided the development and evaluation of the Pregnancy in IBD Decision Aid (PIDA) [21]. Figure 1 outlines the sequence of events in the design and evaluation of the decision aid as recommended by IPDAS. A steering committee was assembled comprising four IBD specialists (VH, AJW, YL, LD), a general gastroenterologist (DS), an obstetrician (FFH), an obstetric medicine physician (RK), a paediatric gastroenterologist (EW), two patient representatives (KB, VL), a shared decision making expert (DK), an information and knowledge translation specialist (KI) and a perinatal pharmacoepidemiologist (MDV). The steering committee conducted regular meetings by teleconference throughout the development process (SC, TH, NK, KOC).

Three study sites were chosen to conduct user testing: 1. Liverpool Hospital (LH), New South Wales, Australia 2. Pacific Gastroenterology (PG), British Columbia, Canada 3. Mt Sinai Hospital (MSH), Ontario, Canada. Focus groups and interviews were conducted to explore patient and clinician views on decisional needs in relation to pregnancy and IBD and to receive feedback regarding methods in which content could be best delivered. Additional guidance regarding reproductive decisional needs in the setting of IBD was obtained from research conducted by VH [14]. An existing systematic review within the field of pregnancy and IBD [9] was updated to ensure the comprehensive inclusion of content, which was subsequently appraised and organised into themes by the steering committee. An electronic prototype of the decision aid with a focus on (a) desires and ‘ideal’ timing for conception and (b) medication choices during pregnancy was produced and then evaluated by users for feasibility across the three sites.

### Phase one: focus groups

Patient and clinician focus groups were conducted at PG to explore issues of concern in pregnancy and IBD. Patient participants were recruited through social media advertising with Crohn’s and Colitis Canada and contacting patients who had previously provided permission to be contacted regarding research opportunities. Clinicians were affiliated with the University of British Columbia across various relevant specialties. Focus groups were moderated by a clinician and recorded and subsequently transcribed for analysis in terms of key themes. In addition, another clinician took fieldnotes to document key discussion items and contextual information. Duration of patient and clinician focus groups were one hour and thirty-five minutes and one hour respectively.

### Phase two: review and synthesize evidence

To identify the most up-to-date evidence on the management of IBD during preconception, pregnancy and postpartum, studies published subsequent to the development of the Toronto Consensus Pregnancy Statements [9] were reviewed. The same search string and selection criteria used in the development of the Toronto Consensus Pregnancy Statements were implemented. MEDLINE and EMBASE were searched from Jan 1, 2014 to Apr 29, 2018. The 2016 Toronto Consensus Statements included publications published in MEDLINE from 1946 to Nov 2014 and in EMBASE from 1974 to Nov 2014. The overlap (from January 2014 to Nov 2014) ensured completeness. In addition, ClinicalTrials.gov was systematically searched from inception to April 29, 2018. The search strategy used for this additional search is presented in section of “Appendix 1”. The synthesis of evidence resulting from the systematic search and the Toronto Consensus Pregnancy Statements was conducted by the members of the steering committee and based on their individual areas of expertise.

### Phase three: decision aid design and evaluation

#### Prototype design

Using content resulting from Phase One and Phase Two, a paper version of the PIDA prototype was drafted, which was then converted into a PowerPoint® version to enable incorporation of clickable logic. Subsequently, there was development of an electronic prototype through utilisation of the digital media company, Tactica.^1^ The current PIDA prototype can accessed at http://ibdpregnancyaid.com/.

The steering committee subsequently provided feedback regarding the design and content of PIDA. An opportunity was offered to formally critique the comprehensibility, usability and accuracy through completion of the Clinician or Patient Feasibility Questionnaire (See section of “Appendix 2a and 2b”). The questionnaire was designed based on tools used in preceding decision aid studies [22–24], with questions pertaining to the time required to review PIDA, perceived readability, content amount, usefulness for the user (if patient user) as well as that anticipated for others, ability to aid with values clarification (if patient user) and accuracy (if clinician user).

#### Patient feasibility testing

To ensure content saturation, feedback regarding the current PIDA prototype was sought from patients at different reproductive stages (preconception, pregnancy, and post-partum) using individual patient interviews and questionnaires. Participants included women of 18–45 years of age with confirmed diagnosis of IBD who (a) had prior pregnancy history (including those within 12 months of delivery, i.e., post-partum), (b) had no pregnancy history but were interested in considering issues surrounding pregnancy, or (c) were currently pregnant. Women who could not speak or read English sufficiently to complete surveys or use the decision aid and those with known previous adverse pregnancy outcomes were excluded. We aimed to have at least four representative participants from each of the reproductive stages of preconception, pregnancy, and post-partum across the three sites for the focus group (Phase One) and interviews (Phase Three) combined. The rationale for this sample size was based on review of previously published decision aids, which included between 15 and 20 participants in their scoping and design phases [22, 25, 26]. The concept of feasibility testing and the associated questionnaire was based on preceding publications focussing on the development of exemplary decision aids [22, 24]. Acknowledging that the initial patient focus group was limited by both size and pregnancy stage (i.e., all participants preconception), the patient interviews were considered of significant importance in the design of PIDA to ensure that appropriate decisional themes and associated content had been identified and included in PIDA.

Patient interviews were conducted by research coordinators or an IBD fellow at one of the three sites. The participant had the opportunity to review the PIDA prototype in the week preceding their interview using the website link. Basic demographic data was collected for each participant at the interview (age, reproductive status and IBD type (UC or CD)). An interview script was designed a priori and used to guide the interview. A template facilitating note taking during the interview was also designed (See section of “Appendix 3”). Each interview took approximately 30 min. In addition to patient interviews, participants completed a Patient Feasibility Questionnaire (See section of “Appendix 2b”). Interviews were analysed using thematic analysis [27] and questionnaire data using descriptive statistics. The compiled feedback obtained was then used to make further changes to the PIDA prototype.

## Results

### Phase one: focus groups

#### Patient focus group

Three patients participated in the focus group, while a further seven who were also interested could not attend on the day due to personal or employment reasons. Median age of participants was 32 years, all with CD and in a preconception stage; one had a history of prior surgery (diverting stoma) for perianal disease and all three were on biologic therapy. Two were currently employed, and the other receiving a disability pension.

The transcript generated from the focus group was analyzed in terms of patient concerns, patient observations as a woman with IBD who is considering pregnancy and patient recommendations for the decision aid and specialist care. Patient concerns regarding conception and pregnancy included (a) the negative impact of active disease on both maternal and fetal/infant health (b) the potential impact of current and past drug therapies on the fetus/infant (c) the ability to care for a child in the setting of being unwell and (d) the ability to conceive, maintain a pregnancy and deliver in the setting of previous abdominal surgery. Recommendations for the design of the decision aid included the ability to facilitate joint decision making (patient and clinician) for decisions surrounding medication management in pregnancy and the promotion of the tool for users at any stage of their reproductive life, including at diagnosis in order for patients to know that pregnancy is an option despite IBD. Exemplary quotes for the expressed concerns and recommendations are shown in section of “Appendix 4a”.

#### Clinician focus group

In attendance at the focus group were two IBD nurses, an obstetrician, neonatal intensivist, two gastroenterologists (IBD Specialists), gastroenterologist (IBD Specialist with expertise in pregnancy) and two IBD fellows.

The transcript generated from the focus group was analysed by identification of key terms, including clinician perception of patient concerns, clinician concerns regarding pregnancy in the setting of IBD, clinician observations as a health care professional for women with IBD and clinician recommendations for the decision aid. Perceived patient concerns included (a) medications in pregnancy, and in particular the potential for birth defects and impact on immunity, (b) infection risk in infants and safety of infant vaccination (c) plan for flares during pregnancy (d) nutrition, (e) contraception and (f) fertility. Recommendations for design of the decision aid included the ability to provide simplified information to patients at multiple stages (for example, preconception and pregnancy) of their reproductive life. Furthermore, the design of the decision aid was perceived as having a role in facilitating discussion with treating specialists, and hopefully promoting opportunities for discussions regarding pregnancy early on in the disease course that may not otherwise have occurred. Exemplary quotes for the expressed concerns and recommendations are shown in section of “Appendix 4b”.

### Phase two: review and synthesize evidence

The literature review identified 306 articles (290 following duplicate removal), with 104 records retained following title and abstract screening. Of the remaining 104 articles, 29 full text articles were included to guide the decision aid content beyond what had been utilised to formulate the Toronto Consensus Pregnancy Statements [11, 28–56].

### Phase three: decision aid design and evaluation

#### Prototype

The decision aid was designed to include a broad range of content, extending from fertility concerns through to post-partum issues and accordingly is considered relevant for users regardless of their reproductive stage. However, the steering committee chose two key decisions based on predominant themes of discussion in the focus groups. Patient interview results also confirmed the perceived importance of the following decisions:

*Desires and ideal timing for conception* The desire to attempt conception and the ideal timing of such was considered in the design of the information presented. Given recognition of the contribution of fears relating to IBD and pregnancy, including the impact of disease activity on pregnancy, concerns regarding medication use, fear of disease inheritance and concerns surrounding delivery, such topics were given emphasis in the design.

*Medication choices during pregnancy* The decision as to what to do with IBD related medications during pregnancy was presented. This was supported by the rationale that medication management is essential during pregnancy (to maintain disease control given disease activity has been associated with adverse pregnancy outcomes) and specific medication information needs to be tailored to preconception, pregnancy and postpartum stages. The presentation of information included numerical probabilities, such as that relating to the impact of active disease on adverse pregnancy outcomes. Values regarding medication usage during pregnancy were also assessed prior to and following presentation of the aforementioned information.

Four clinicians and two patient representatives from the steering committee provided formalised feedback, including an Obstetric Physician, an Obstetrician and an adult and a paediatric Gastroenterologist (section of “Appendix 2c”). Feedback indicated adequacy of length, readability, content amount and values assessment, in congruence with the feedback that PIDA is a useful tool. It is noted that the patients from the steering committee were already well educated on pregnancy and their IBD in the context of previous pregnancies and prior physician education, and hence it was reported that the decision aid did not personally impact their understanding and decision making.

Following several iterations, a prototype was agreed upon which was deemed suitable for alpha testing. (See section of “Appendix 5” for exemplary section of prototype) Reading level was assessed using the Flesch Kincaid index [57]. Four representative content sections were chosen from the prototype for testing—disease activity, nutrition, substance abuse and post-partum medications. The obtained reading levels ranged between an average grade level of 13–16, deemed able to be read easily by 18–19 year olds and 21–22-year olds respectively.

#### Patient feasibility testing

##### Patient interviews

Thirteen patients across three sites were interviewed, either in person at the institutional site or via telephone. Median age of participants was 31 years (interquartile range (IQR) 30.25–33), six with UC and seven with CD. Three were in preconception, six in pregnant and four in post-partum stages. For nine of these patients, expanded demographic data was available. (See Table 1).

**Table 1:** Demographic variables of feasibility testing participants (n = 9)

Demographic variable	Frequency of demographic n (%)
Age (median years + IQR^a^)	31 (29.5–33.5)
Ulcerative colitis	4 (44)
Crohn’s disease	5 (56)
Duration of disease (median years + IQR)	5.5 (3.5–13)
*Current medications*	
5-aminosalicylates	3 (33)
Corticosteroids	2 (22)
Immunomodulator (Thiopurine)	4 (44)
Biologics	4 (44)
Anti-tumour necrosis factor	4
Vedolizumab	0
Ustekinumab	0
*Surgical history*	
Yes	2 (22)
No	7 (78)
*Pregnancy stage*	
Preconception	3 (33)
Pregnancy	4 (44)
Post-partum	2 (22)
*Currently breastfeeding*	
Yes	2 (22)
No	0
Not applicable	7 (78)
*Prior pregnancies (if pregnant, excludes current)*	
Yes	5 (56)
No	2 (22)
*Marital status*	
Married	6 (67)
Common-law	1 (11)
Single	2 (22)
*Highest level of education*	
High school diploma	2 (22)
Trade, technical, vocational, business school	1 (11)
University undergraduate degree	3 (33)
Post graduate degree	3 (33)
*Total income (CAD/AUS $)*	
20,000–39,990	1 (11)
40,000–69,900	1 (11)
70,000–99,000	2 (22)
100,000+	5 (56)

^a^IQR: Interquartile

The thematic analysis of the interviews revealed that participants’ most desired content related to medication management during conception, pregnancy and lactation. Additional pregnancy in IBD questions related to other topics such as fertility, inheritance and delivery. Feedback regarding PIDA was predominantly positive, with comments pertaining to adequacy of content coverage, personalization, readability and unbiased information presentation. Suggestions were made for enhancement of design and inclusion of further content. Design related suggestions were the inclusion of visual aids, a summary page and the availability of links to further information, all of which have now been incorporated into the PIDA prototype. Recommendations for content additions which have since been incorporated into the current prototype included statistical representation of inheritance, exercise recommendations, pregnancy related gastrointestinal symptoms and differentiation from IBD symptoms and the timing of recommencement of medications post-partum. Content to be included in subsequent prototype iterations include the impact of IBD on sexual function, expected laboratory changes during pregnancy, and additional post-partum issues including IBD activity and newborn care. The responses to interview questions are summarised and further exemplified in Table 2.

**Table 2:** Main themes emerged from patient interviews

**Main concerns** The health of fetus/infant Effect of IBD^a^ medications on pregnancy, fetal, and neonatal outcomes and their safety during breastfeeding IBD and Delivery
**Main information needs** When is the ideal time to become pregnant when you have IBD? How does my IBD effect my fertility? Will I be able to breastfeed with IBD? Can I have a vaginal delivery? Will I pass IBD or my immune system to my baby? Will any of my IBD drugs pass through to my baby? (during pregnancy & breastfeeding)
**Feedback on PIDA** Quantity of information on the slides was not overwhelming Nothing seemed to be missing or too elaborate Information was presented in a neutral light
**Suggested improvements to presentation or content** Pictures and diagrams to help visualize information Statistics for example, likelihood of IBD inheritance and flares Summary page and links to further information Suggestions how to improve communication between specialists Sexual function and how it is impacted by IBD Pregnancy related gastrointestinal symptoms vs IBD related symptoms Laboratory changes during pregnancy Safety or recommendations for exercise during pregnancy Analgesia during delivery Any special things for adjusting to home life in the presence of IBD

^a^IBD: Inflammatory Bowel Disease

##### Feasibility questionnaire

Feasibility questionnaires were completed at two of three sites. Scoring indicated that length was considered adequate, with a median time of 15 min (IQR: 10–16.25) for review. Similarly, readability and content amount were both scored as appropriate. Patients reported that the decision aid was useful in terms of obtaining information and decision making and noted that they would recommend to others in their situation. Importantly, it was indicated that PIDA enabled thorough assessment of patient values. Numerically there did not appear to be substantial variation between responses from participants who were pregnant as opposed to preconception or post-partum. Summarised feasibility questionnaire responses are displayed in Table 3.

**Table 3:** Patient feasibility questionnaire responses (n = 9)

Question statement	Response (Median)
Time for review of decision aid (minutes + IQR^b^)	15 (10–16.25)
Length^a^ (where 3 indicates adequate, 1 short and 5 excessive)	3
Readability^a^ (where 3 indicates appropriate, 1 simplified and 5 challenging)	3
Content amount^a^ (where 3 indicates appropriate, 1 limited and 5 excessive)	3
Usefulness for patient understanding and decision making^a^ (where 3 indicates no impact on understanding and decision making, 1 confusing, and 5 useful)	5
Recommending the decision aid to others in my situation^a^ (where 3 indicates suggested, 1 not recommended and 5 highly recommended)	5
Patient values^a^ (where 3 indicates adequate assessment of patient values, 1 inadequate and 5 very well)	5

^a^Likert scale of 1–5

^b^IQR: interquartile range

## Discussion

There has been increasing recognition of the importance of tailored IBD management during conception, pregnancy and postpartum phases to optimise obstetric and infant outcomes. This has been parallel to the increasing complexity of therapeutic options for IBD. Fortunately, accompanying this is an increasing volume of data providing reassurance for the safety during conception, pregnancy and lactation of most medications prescribed for IBD. However, there remains deficiencies in clinician and patient education regarding the management of IBD during pregnancy. This has been highlighted in previous studies demonstrating high rates of voluntary childlessness, inappropriate medication management and the recognised desire for further education from both interest groups [3, 4, 20, 58].

Accordingly, we have embarked on the development of a personalised decision aid to help meet the aforementioned gap in patient education, which has been further motivated by preceding evidence for the use of decision aids in pregnancy [16]. To guide this process, the IPDAS guidelines have been followed [21]. In addition, the Standards for UNiversal Reporting of patient Decision Aid Evaluations (SUNDAE) checklist was utilised to prepare the reporting of the design process and results [21, 59]. The novelty of PIDA is that it is the first interactive personalized decision aid for pregnancy in IBD. Other available online resources to date are information presenting, or provide checklists, but none are as interactive or personalized to the extent that PIDA has been designed. We feel this advancement in the field will allow more preconception and pregnant women with IBD to obtain core information that they can use to make informed decisions and/or to stimulate discussion with their clinicians.

Reflecting on discussion and feedback occurring during focus groups and individual patient interviews highlighted the consistent theme of the potential for voluntary childlessness, with contributing factors of fear, limitations in existing knowledge and both individual and community misperceptions. Similarly, another persistent theme was that of medication uncertainty across all stages of reproduction (preconception, pregnancy and post-partum). Accordingly, two key decisions were identified (1) the decision regarding the possibility and timing of conception and (2) the decision around the choice of medications in the peri-partum period. Information relevant to both decisions (such as medication safety in conception, pregnancy and lactation; placental transfer and implication for infant vaccinations and importance of disease activity control) were provided in the decision aid. Questions were incorporated to help assist the individual user to clarify their values with regards to medication related decisions. Given the reporting of patient desires for proactive reproductive counselling in their IBD management (e.g., from the time of diagnosis), it is envisioned that the inclusion of values assessment could prompt PIDA users to consider reproductive decisions earlier in their disease course and potentially further assist in addressing voluntary childlessness.

While there was an attempt to obtain a broad patient perspective in the design and preliminary evaluation process for PIDA, note is made of certain demographic biases, related to the intrinsic difficulties with recruitment, especially within the cohort of young patients who often have additional time constraints related to family (particularly given the involvement of young mothers with children) or professional commitments. Furthermore, it is acknowledged that the content included intensely personal issues, with discussion potentially being further challenged in the setting of an outpatient clinic location. Accordingly, there was a limitation of the number of participants able to attend the initial patient focus group and a decision made not to attempt for conduct further focus groups due to recruitment challenges. Further limitations were the homogeneity of disease type (CD) and preconception status of all participants, however there was inclusion of the impact of a previous IBD surgical history. Given the limitation of focus group size and the desire of participants to be involved in the study at a more convenient location (for example from home), feasibility testing included the option of telephone interviews conducted by the research team. In the future, there could be consideration of videoconference as an alternative method to enhance participant involvement and comfort. It is also observed that the majority of participants in patient interviews were of a high socioeconomic background, and thus feedback obtained may not have been reflective of the intended overall target audience for PIDA, including those with limited reading skills. Future consideration of the potential influence of religious and cultural beliefs on pregnancy related perceptions is also necessary to enhance the generalisability of the decision aid.

Subsequent iterations of the current prototype will enable further fulfilment of the requirements in the criteria for judging quality of decision aids as listed in the IPDAS guidelines [60]. In future prototypes, values questions assisting decision making surrounding the desires and timing of conception will be included. It is also intended that there will be the ability to enable the user to search for keywords, while content will also be presented in additional modes other than written text and graphs (for example, audio or video). Medication content will be expanded, in addition to being colour coded according to compatibility of use in conception, pregnancy and lactation. Additional content inclusion such as the impact of IBD on sexual function and the potential effect of IBD during the post-partum period will occur. Evaluation of the decision aid with patient and clinician alpha testing (including the assessment of the impact of PIDA on the quality of the decision-making process, as well as the decision) will guide future iterations. Furthermore, subsequent beta testing (with a randomised controlled trial) is necessary prior to routine use and promotion of the decision aid. Beyond beta testing, adaptation of the decision aid into different electronic technologies, including that of a mobile applications or video representation, could be considered.

## Conclusions

Given the efforts employed to systematically develop the decision aid thus far, and the favourable initial user feedback obtained, we anticipate that PIDA will be able to meet an unmet need in the education of patients with IBD who are likely to encounter decisions regarding conception, pregnancy and post-partum timing and management. We envision that there may be the potential for minimisation of voluntary childlessness, as well as optimization of maternal, foetal and infant outcomes related to the enhancement of pregnancy-specific IBD management through the use of PIDA.

## Data Availability

The datasets used and/or analysed during the current study are available from the corresponding author on reasonable request.

## Abbreviations

IBD: Inflammatory bowel disease
PIDA: Pregnancy in IBD decision aid
CD: Crohn’s disease
UC: Ulcerative colitis
IPDAS: International Patient Decision Aid Standards
LH: Liverpool Hospital
PG: Pacific Gastroenterology
MSH: Mt Sinai Hospital
UNSW: University of New South Wales
UBC: University of British Columbia
U of T: University of Toronto
U of A: University of Alberta
IQR: Interquartile range
SUNDAE: Standards for UNiversal Reporting of patient Decision Aid Evaluation

## Footnotes

1 Tactica specializes in cross-platform digital media strategy and products for researchers, agencies, and producers. Such products have included numerous health projects, including the HOPE digital platform designed and validated for antenatal and post-partum depression [61].
